# Modified Low Density Lipoprotein and Lipoprotein-Containing Circulating Immune Complexes as Diagnostic and Prognostic Biomarkers of Atherosclerosis and Type 1 Diabetes Macrovascular Disease

**DOI:** 10.3390/ijms150712807

**Published:** 2014-07-21

**Authors:** Alexander N. Orekhov, Yuri V. Bobryshev, Igor A. Sobenin, Alexandra A. Melnichenko, Dimitry A. Chistiakov

**Affiliations:** 1 Laboratory of Angiopathology, Institute of General Pathology and Pathophysiology, Russian Academy of Medical Sciences, Moscow 125315, Russia; E-Mails: a.h.opexob@gmail.com (A.N.O.); igor.sobenin@gmail.com (I.A.S.); zavod@ifarm.ru (A.A.M.); 2 Institute for Atherosclerosis Research, Skolkovo Innovative Center, Moscow 143025, Russia; 3 Faculty of Medicine, School of Medical Sciences, University of New South Wales, Kensington, Sydney, NSW 2052, Australia; 4 School of Medicine, University of Western Sydney, Campbelltown, Sydney, NSW 2560, Australia; 5 Laboratory of Medical Genetics, Russian Cardiology Research and Production Complex, Moscow 121552, Russia; 6 Department of Medical Nanobiotechnology, Pirogov Russian State Medical University, Moscow 117997, Russia; E-Mail: dimitry.chistiakov@lycos.com

**Keywords:** atherosclerosis, atherogenesis, immune complexes, low-density lipoproteins, inflammation

## Abstract

In atherosclerosis; blood low-density lipoproteins (LDL) are subjected to multiple enzymatic and non-enzymatic modifications that increase their atherogenicity and induce immunogenicity. Modified LDL are capable of inducing vascular inflammation through activation of innate immunity; thus, contributing to the progression of atherogenesis. The immunogenicity of modified LDL results in induction of self-antibodies specific to a certain type of modified LDL. The antibodies react with modified LDL forming circulating immune complexes. Circulating immune complexes exhibit prominent immunomodulatory properties that influence atherosclerotic inflammation. Compared to freely circulating modified LDL; modified LDL associated with the immune complexes have a more robust atherogenic and proinflammatory potential. Various lipid components of the immune complexes may serve not only as diagnostic but also as essential predictive markers of cardiovascular events in atherosclerosis. Accumulating evidence indicates that LDL-containing immune complexes can also serve as biomarker for macrovascular disease in type 1 diabetes.

## 1. Introduction

Atherosclerosis results in the inner layer of the arterial wall and is characterized by local lipid accumulation and excessive production of collagen [1,2,3]. Atherosclerosis and atherosclerotic disease (myocardial infarction, stroke, coronary heart disease, and sudden death) are the dominant cause of death in industrialized countries [4].

The markers generally measured for diagnosing of atherosclerosis are lipid parameters of the blood, in particular cholesterol [5]. Even though the direct link between the reduction of cholesterol and the regression of atherosclerosis is not unarguably established [6,7,8], cholesterol is still the key target parameter in the existing anti-atherosclerotic therapy [9]. It is worth noting here that the obtained data about a relation of apolipoproteins to risk scores led to slight improvement in cardiovascular disease risk prediction [10].

It is well known that atherosclerosis develops asymptomatically affecting the intima which is non-innervated part of the arterial wall. Currently, asymptomatic (preclinical) atherosclerosis is not an object of diagnostics and treatment while the number of clinical events on the background of asymptomatic atherosclerosis is quite high [11,12,13]. Despite the advances in screening for preclinical atherosclerosis [14], screening for preclinical atherosclerosis is used, very rarely, in clinical practice.

A search for new reliable biomarkers of atherosclerosis is an important task [15]. This review highlights the accumulated information about low density lipoprotein (LDL) modification naturally occurring in the blood of patients, as well as about circulating LDL-containing immune complexes which emerge to be diagnostic and prognostic biomarker of atherosclerosis and macrovascular disease in type 1 diabetes.

## 2. Low Density Lipoprotein

Lipid deposition in the arterial wall is widely recognized as the earliest pathogenic event in preclinical atherogenesis [16]. Atherogenic lipids enter the arterial intima from the bloodstream and represent multiple modified low-density lipoproteins (LDL) particles [16,17,18]. Lipoproteins, which are mainly constituted from glycoproteins and lipids, are involved in transferring fats through blood and interstitial fluids. Lipoprotein particles have a highly hydrophobic core enriched by hydrophobic tails of phospholipids, fatty acids, cholesterol, and apoproteins. The presence of the hydrophilic core provides the possibility to accumulate esterified cholesterol and triglyceride molecules within each particle and then carry those to the target tissue. The apoprotein molecule is essential for stabilizing structure of the lipoprotein particle and is responsible for interaction with fat-metabolizing enzymes and cell surface receptors in order to distinguish which lipid molecules should be removed or added to the particle [18,19,20].

LDL is a lipoprotein fraction of particles that usually ranges in size of 18–28 nm and density of 1.019–1.063 g/mL [21]. However, LDL particles, having a 30 nm and larger size, have been detected [22]. LDL particles are generated in the bloodstream during the metabolic processing of apolipoprotein B-100 (apoB-100)-containing lipoproteins [23]. The lipoprotein processing starts from the liver secretion of very low density lipoprotein (VLDL) particles, which then become the subject of multiple transformations mediated by various serum lipolytic enzymes and lipid transporters before the final formation of LDL particles [24].

The LDL particle contains a single apolipoprotein B-100 (apoB-100) molecule, 80–100 molecules of secondary proteins, approximately 3000 molecules of linoleic acids, 1500 molecules of esterified and non-esterified cholesterol (in average), and variable numbers of triglycerids and phospholipids composed mainly of phosphatidylcholine and sphingomyelin [25]. In a distinct LDL particle, ApoB-100 plays a central role by stabilizing and maintaining its structure and composition [26]. ApoB-100 is a large glycoprotein comprising 4536 amino acids and 24 potential *N*-glycosylation sites. This protein is highly hydrophobic and hence remains with the lipoprotein particle throughout the metabolism [27]. ApoB-100 has an α-helical content of 25% and approximately 50% β-sheet structure, with five large lipid-associating domains [28]. For the human ApoB-100 molecule, Segrest *et al.* [29,30] suggested the pentapartite structure: NH_2_-α_1_-β_1_-α_2_-β_2_-α_3_-COOH, showing the presence of two regions of amphipathic β-strands alternating with two regions of amphipathic α-helices and the third *N*-terminal amphipathic α-helical domain.

In human serum, two major apoB isoforms (apoB-100 and apoB-48) exist. The apoB-48 isoform is produced after RNA editing of the apoB-100 transcript at residue 2180 resulting in the creation of a premature stop codon [31]. Therefore, the two isoform share the common *N*-terminal domain. However, apoB-48 is not able to bind to the LDL receptor since it lacks the LDL receptor-binding domain. The α_3_ α-helical region comprising 11% of apoB-100 is mobile and is involved in the control of availability of the LDL receptor-binding domain during the conversion of very low density lipoproteins (VLDL) to LDL in blood plasma [32]. The LDL receptor-binding domain contains three proline-rich clusters, which are exposed on the LDL surface and essential for protein-protein interactions [33]. The first 1000 residues of human apoB-100 (*i.e.*, the entire α_1_α-helical region and 200 first amino acids of the β_1_ domain) were shown to form a three-dimensional structure that is similar to that of lipovitellin, an egg yolk lipoprotein containing a “lipid pocket” [34]. This “pocket” is involved in binding microsomal triglyceride transfer protein to create a lipid transfer pocket required for assembly of the apoB-containing lipoprotein particle [34].

LDL receptors located on the surface (in clathrin-coated pits) of target cells are responsible for specific binding LDL particles followed by internalization of LDL-cholesterol through the mechanism of endocytosis [35]. Hepatic LDL receptors are primarily responsible for withdrawal of LDL particles from the circulation, thus, tending to ensure that serum LDL levels remain at a physiologically relevant range. However, increased serum LDL concentrations diminish the functioning LDL receptor-dependent pathway and promote the influx of LDL particles into the arterial wall where they become trapped and modified thereby be converted to the key players in the vicious circle of proatherogenic inflammation and lipid accumulation [18,36].

Apart from transferring lipids, human VLDL and LDL are also suggested to be involved in intra-organismal protein transfer and delivering proinflammatory and prothrombotic protein mediators from the sites of synthesis to inflamed and embolic destinations [37,38]. For example, a recent proteomic analysis revealed presence of 95 VLDL- and 51 LDL-associated proteins respectively [39]. Along with all known apolipoproteins and lipid transport proteins, lipoprotein particles were shown to contain coagulation proteins, components of the complement system, and antimicrobial-including prenylcysteine oxidase 1, dermcidin, cathelicidin, tissue factor pathway inhibitor-1, and fibrinogen α chain [39]. Human diseases related to LDL-associated proteins could involve dyslipidaemia, coagulation disorders, atherosclerosis, and other vascular pathology. In pathologic conditions, protein composition of LDL could be significantly different from that of LDL in normal serum. LDL-apheresis treatment used for reducing serum LDL cholesterol levels and preventing acute cardiovascular events in homozygous patients with familial hypercholesterolemia were found to remove up to 48 types of proteins including procoagulation and thrombogenic factors, complement factors, inflammatory mediators, and adhesion molecules [40]. Interestingly, serum LDL derived from healthy humans contain several non-traditional apoproteins, such as serum amyloid A-IV [41], a biomarker of acute inflammation, whose content could be markedly increased in all lipoprotein fractions, especially in LDL from atherosclerotic patients [42]. Serum amyloid A-IV is released by liver in response to proinflammatory injury and is thought to display a variety of proatherogenic effects including endothelial dysfunction [43], foam cell formation, and induction of proinflammatory cytokines in macrophages [44].

A single apoB molecule accounts for approximately 25% while lipids contribute for the remaining 75% of the molecular weight of the LDL particle [45]. The hydrophobic lipid core consists of cholesterylester and triglyceride molecules, which make up more than 40% of particle mass. The core is surrounded by the phospholipid monolayer corresponding to about 20% of particle mass. Varying amounts of free cholesterol are incorporated in the shell and the core regions accounting for 15% of particle mass [45]. In the LDL particle, an additional hydrophobic interfacial layer composed of phospholipid acyl chains, free cholesterol, some cholesteryl ester molecules, and hydrophobic protein domains are found thereby reflecting the interplay between neutral core lipids and the surface layer [25].

LDL could be considered as a dynamic construct that needs to respond to changing environmental conditions during lipid exchange. Indeed, during particle remodeling, apoB and the surface phospholipids must rearrange to compensate for changes in the surface area and surface pressure [46]. Within the interfacial layer, lipids are not homogeneously distributed but form local microenvironments [25]. For example, in the LDL particle, two regions one enriched with sphingomyelin and free cholesterol, the other one rich in phosphatidylcholine and poor in free cholesterol were identified. The latter was shown to be associated more closely with the apoB-100 molecule [25,47]. Recent 3D-images show that LDL represents discoidal-shaped particle with two flat surfaces on opposite sides. In this model, apo B100 encircles LDL at the edge of the particle, while the phospholipid monolayer is rather located at the flat surfaces, which are parallel to the cholesteryl esters layers in the core [48]. LDL particles share a common feature: The cholesteryl ester molecules in the core undergo a structural transition from an ordered liquid-crystalline phase to a liquid oil-like state depending on temperature and chemical composition [49]. The transition temperature is close to body temperature and inversely correlates to the content of triglycerides within the lipid core [50].

To date, over 350 various lipid species from 19 lipid subclasses were found in human LDL [51]. Phosphatidylcholine is the most abundant phospholipid in serum lipoproteins including LDL. Phosphatidylcholine is a major constituent of cell membranes. This phospholipid is also involved in membrane-mediated cell signaling and phosphatidylcholine transfer protein (PCTP) activation of other enzymes [52]. In the liver, active phosphatidylcholine biosyntesis is required for VLDL secretion [53]. Since cholesterol prefers to interact with phosphatidylcholine and sphingomyelin, both phospholipids are essential for blood transport of cholesterol and cholesterylesters by LDL and other serum lipoproteins [54]. Recently, intestinal microflora was shown to metabolize dietary phosphatidylcholine to choline, trimethylamine *N*-oxide (TMAO), and betaine, e.g., to catabolites that increase risk of atherosclerosis and cardiovascular disease [55].

Sphingomyelin is an essential structural component of serum lipoproteins and the second major phospholipid after phosphatidylcholine. Sphingomyelin is a prevalent sphingolipid in membranes of mammalian cells and this lipid class is specifically enriched in the plasma membrane, the endocytic recycling compartment, and the *trans* Golgi network. Sphingomyelin is involved in the regulation of endocytosis and receptor-mediated ligand uptake, in ion channel and G-protein coupled receptor function, in protein sorting, and function as receptor molecules for bacterial toxins and non-bacterial pore-forming toxins [54].

In inflammatory conditions such as atherosclerosis, proinflammatory mediators stimulate secretion of Zn^2+^-dependent sphingomyelinase by endothelial cells and macrophages that hydrolyses LDL sphingomyelin to ceramide (*N*-acetyl-d*-*sphingosine) [56]. Ceramide could be also generated in the liver by biosynthesis from serine and palmytoil-coenzyme A and then secreted to the bloodstream in the form of VLDL [57]. In the serum, ceramide-enriched VLDL could be then converted to ceramide-enriched LDL. The ceramide content inversely correlates with the sphingomyelin content in serum lipoproteins. In humans, VLDL and LDL are especially enriched with ceramide and dihydroceramide while HDL contain low amounts of these sphingolipids [58]. Physiologically, ceramide serves as an inductor of multiple stress responses initiated by proapototic and proinflammatory agents [59].

However, enrichment of LDL with ceramide is highly proatherogenic. Increase in LDL ceramide was found to increase the aggregation rate of LDL particles [60], to enhance arterial matrix remodeling [61], and to induce foam cell formation [62]. The increased conversion of LDL sphingomyelin to ceramide may increase the vulnerability of LDL for oxidation [57]. Ceramide-enriched LDL can be taken up by the endothelial cells in a receptor-mediated fashion and can deliver excess ceramide to the cells [63]. Ceramide was shown to activate reactive oxygen species (ROS), mitochondrial oxidative damage, and apoptosis in vascular cells [64]. Ceramide could enhance inflammation through own metabolites and signaling molecules such as sphingosine and sphingosine-1-phosphate [65,66].

Electronegative LDL is a minor subfraction of modified LDL that is normally present in circulation [67] (Sánchez-Quesada *et al.*, 2004). Compared to native LDL, electronegative LDL shows some proatherogenic characteristics including increased content of lysophosphatidylcholine (LPC) and presence of phospholipase C (PLC)-like activity of unknown origin [46,68]. The PLC-like activity in electronegative LDL hydrolyzes LPC, sphingomyelin, phosphatidylcholine, and other choline-containing phospholipids with formation of phosphocholine, ceramide, monoacylglycerol (MAG), and diacylglycerol (DAG) [69]. While hydrophilic phosphocholine leaves the LDL particle hydrophobic molecules of ceramide, MAG, and DAG retain in the LDL particles and increase their aggregation through enhancing hydrophobic contacts [69]. In addition, these lipids induce proinflammatory properties of electronegative LDL [68]. DAG activates protein kinase C and adenylcyclase, which generates cAMP, a key molecule in many biological processes [70]. DAG is also required for propagation of the downstream signals needed for activation of NF-κB, a proinflammatory transcription factor.

## 3. LDL in Atherosclerosis

The entrance and accumulation of free cholesterol in the arterial wall are crucial events in early atherosclerosis [1,16]. Compared to other plasma lipoprotein fractions, LDL particles are especially enriched with non-esterified (so called free) cholesterol that can account up to 50% of the particle weight [26,71]. LDL particles are the main vehicles responsible for cholesterol transport. The proatherogenic value of high cholesterol content in the LDL fraction and its possible significance in predicting cardiovascular risk was suggested in early longitudinal epidemiological studies such as the Framingham Study [72]. Finally, in 2008, the American Diabetes Association (ADA) and the American College of Cardiology (ACC) recommended quantification of LDL particle content by nuclear magnetic resonance spectroscopy as essential for accessing individual risk of cardiovascular events [73].

In fact, total cholesterol and high-density lipoprotein (HDL) cholesterol seems to be the best predictor of the cardiovascular risk to date. Replacement of these parameters with a combination of lipid-related markers such as apoB and apoA-I, lipoprotein (a), or lipoprotein-associated phospholipase A2 does not improve cardiovascular disease (CVD) prediction but adding these markers to the combination of total cholesterol and HDL cholesterol slightly strengthen the prediction power [10]. Although some studies reported that apoB is superior to total cholesterol or LDL cholesterol in predicting CVD risk [74,75], there is a serious problem in the reproducibility and standardization of measuring apoB due to the significant size and epitope heterogeneity of this molecule [76]. Overall, compared with separate lipid-related CVD markers, HDL cholesterol appears to be better correlated with atherogenic lipoproteins and other cardiovascular risk factors because high HDL cholesterol itself strongly protects against CVD, while low HDL cholesterol is inversely correlated with levels of atherogenic lipoproteins and is associated with several cardiometabolic risk factors [77,78].

Although LDL binds to LDL receptor and oxidized LDL (oxLDL) binds to scavenger receptor, the vascular effects of minimally modified LDL and oxLDL are very similar [79]. Both of these derivatives activate endothelial cells, vascular smooth muscle cells (VSMCs), and monocytes, and enhance vasoconstriction, thrombosis, and platelet aggregation. Furthermore, in atherosclerotic vessels, increased LDL levels stimulate endothelial expression of adhesion molecules and chemokines such as vascular cell adhesion protein-1 (VCAM-1), intercellular adhesion molecule-1 (ICAM-1), and monocyte chemoattractant protein-1 (MCP-1) [80]. In the vascular endothelium, LDL also decrease production of such nitric oxide (NO), an important vasodilator, and stimulate secretion of the vasoconstrictor endothelin-1 [81,82,83]. In VSMCs, LDL and their derivatives activate production of proinflammatory cytokines and growth factors, particularly platelet-derived growth factor (PDGF), and procoagulant factors such as tissue factor or plasminogen activator inhibitor-I (PAI-I) [83]. Indeed, elevated serum LDL levels induce proinflammatory and procoagulant phenotype in vascular cells thereby promoting endothelial dysfunction and contributing to proatherogenic vascular changes.

In the pathologic conditions such as oxidative and metabolic stress, hyperlipidemia and/or diabetic hyperglycemia, circulating and intraintimal LDL are subjected to multiple enzymatic and non-enzymatic chemical modifications [17,84,85]. The modified LDL but not native (non-modified) LDL is accumulated in arterial cells. Fat-laden resident smooth muscle cells and macrophages are unable to utilize engulfed modified LDL and transform into so-called foam cells. The accumulation of foam cells in the arterial wall causes formation of initial lesion and then fatty streaks that actually represent early lesions in proatherogenic progression [86]. In addition, intracellular accumulation of modified LDL is cytotoxic for resident cells and macrophages and hence initiates inflammatory response against apoptotic and necrotic cells [87,88].

Modified LDL also possesses immunogenic properties and induces formation of autoantibodies [89,90] that further contribute to arterial inflammation. In overall, IgG self-antibodies to modified LDL are associated with proatherogenic properties whereas IgM self-antibodies to LDL with atheroprotective properties [91]. The presence of proatherogenic modified LDL and circulating LDL-containing immune complexes in blood of patients affected with atherosclerosis can explain the phenomenon of atherogenicity, *i.e.*, ability of blood sera from affected subjects to induce proatherogenic changes in the phenotype of cultured human aortic resident cells, monocytes, and macrophages [92]. These antibodies and immune complexes have immunomodulatory properties and hence are able to modulate proatherogenic inflammation.

LDL-specific antibodies and their immune complexes with LDL could be detected not only in atherosclerotic plaques, but even in blood of apparently healthy children and newborns suggesting that proatherogenic risk factors may occur early in life [89,93,94]. Indeed, identification of these circulating components in blood may have diagnostic and prognostic value for patients with coronary artery disease (CAD). In this review, we consider mechanisms of formation of proatherogenic modified LDL and immune complexes between self-antibodies and modified LDL and characterize their significance as prognostic and diagnostic markers of atherosclerosis.

## 4. LDL Modifications

### 4.1. Oxidized LDL

LDL modifications and especially oxidation may play a key role in induction of atherogenesis. Non-oxidized LDL does not accumulate in macrophages while modified LDL does [95]. OxLDL is not able to bind to the LDL receptor and start to be taken up by arterial cells. To date, the precise mechanisms of LDL oxidation are not fully understood although several possible mechanisms have been suggested. Those include oxidation mediated by ROS generated by monocytes, macrophages, and endothelial cells, action of metal ions, and enzymatic reactions catalyzed by lypoxygenase and myeloperoxidase [96]. ROS, lypoxygenase, and metals (Fe^3+^, Cu^2+^) are preferentially involved in oxidation of lipid constituents of LDL whereas myeloperoxidase and hypochlorous acid (HOCL) oxidize apoB-100 [97]. Myeloperoxidase, an oxidase produced by neutrophils and macrophages, generates HOCL and hypothiocyanous acids (HOSCN), both are potent oxidants that in turn can modify the apoB-100 molecule in multiple sites [98]. In oxLDL, the content of antioxidants such as coenzyme Q10, tocopherols, β-carotene, and lycopene, was 1.5- to 2-fold lower than in native LDL suggesting for a higher susceptibility of oxLDL to be further oxidized [99].

OxLDL can trigger inflammation through several mechanisms. In macrophages and monocytes, oxLDL induces fat deposits, ROS production, proinflammatory responses, and apoptosis [100,101]. In macrophages, OxLDL are able to target several scavenger receptors including CD36 and the receptor for advanced glycation end-products (RAGE) that then induces production of proinflammatory cytokines such as tumor necrosis factor (TNF)-α, oxidative stress, and enhances chemotaxis [101].

In endothelial cells, oxLDL stimulates expression of lectin-like oxLDL receptor-1 (LOX-1), which is up-regulated in atherosclerotic plaques and is implicated in several pathological processes that control lesion progression [102]. OxLDL activates production of interlerleukin-8 (IL-8) by endothelial cells, a chemokine that attracts inflammatory cells to the site of inflammation, increases migration of smooth muscle cells (SMCs) of the tunica media to the intima, and activates TNF-α production in monocytes/macrophages [103]. OxLDL could also initiate secretion of IL-8 in aortic SMCs through activation of ROS-mediated signaling [104].

Several studies supported good predictive and diagnostic value of oxLDL measured by a specific monoclonal antibody for atherosclerosis-related events including coronary and cerebral vascular disease. In a follow-up study of 326 clinically healthy Swedish men, Wallenfeldt *et al.* [105] showed association between plasma oxLDL levels measured by a specific monoclonal antibody, plaque size and numbers, and carotid artery intima-media thickness (IMT) after adjustment for other cardiovascular risk factors suggesting that circulating oxLDL measured by a specific monoclonal antibody may serve as a prognostic marker of subclinical atherosclerosis. In line with this, Chen *et al.* [106] observed correlation between oxLDL levels measured by antibodies and carotid artery IMT in healthy Taiwanese, thereby providing evidence that measuring oxLDL concentration can have prognostic value for preclinical atherosclerosis of the carotid artery. Furthermore, individuals with low levels of anti-oxLDL antibodies and highest oxLDL content measured by antibodies had the highest risk of carotid atherosclerosis [106]. Overall, elevated levels of oxLDL measured by antibodies have been shown to be associated with increased relative risk (RR) of cardiovascular events ranging from 1.9 and 3.2 after adjustment for various potential confounders [107]. Except for preclinical atherosclerosis, oxLDL levels measured by antibodies may be predictive for clinically manifested atherosclerosis, acute coronary syndromes, and plaque vulnerability [108,109,110,111].

### 4.2. Malondialdehide LDL

ROS degrade polyunsaturated lipids forming malondialdehide (MDA) [112]. Indeed, since LDL are enriched with polyunsaturated linoleic acid, oxidation of this fatty acid may generate MDA. In fact, MDA represents an advanced lipooxidation endproduct that is widely recognized as a biomarker of oxidative stress [113]. When less than 15% of the lysine residues of human apoB-100 are modified by MDA, LDL is able to bind to the LDL receptor. However, if more than 15% of the lysine residues are MDA-modified, the LDL receptor fails to bind LDL and LDL intake starts to be mediated by a scavenger receptor [114]. In the apoB-100 molecule, the *N*-terminus was shown to be essential for recognition of malondialdehide LDL (MDA-LDL) by a scavenger receptor of human monocytes/macrophages followed by receptor-mediated uptake of modified LDL [115].

In Japanese patients with CAD, a positive correlation between MDA-LDL levels measured by the ELISA method and coronary artery IMT and an inverse correlation between MDA-LDL and size of LDL particles was observed suggesting for association with coronary atherosclerosis [116,117]. The greatest content of MDA-LDL in small dense LDL fraction that itself is highly proatherogenic [118] may suggest that small dense LDL are preferentially subjected to the MDA-mediated oxidation [119]. However, recent studies (that will be considered below) suggest that levels of MDA-LDL-containing immune complexes have a better predictive value for atherosclerosis-related diseases.

### 4.3. Glycated LDL

In diabetic hyperglycemia, LDL can be intensively and irreversibly modified *via* mechanisms of non-enzymatic glycation and glycooxidation [120]. In the LDL particle, both the lipid and protein (apoB-100) moieties are the targets for glycation. In non-diabetic patients, up to 4.8% of total apoB can be glycated whereas the percentage of glycated apoB can account up to 14.8% of total apoB in type 2 diabetic subjects [121]. Small-dense LDL are especially prone to glycation in type 2 diabetes and metabolic syndrome [122]. Glycated LDL in turn became more sensitive to further oxidation. Analysis of LDL subfractions derived from the blood of diabetic patients revealed the presence of a highly proatherogenic small-dense modified LDL subfraction enriched with glycated and desialylated LDL [123,124] and capable to increase cholesterol uptake in vascular cells derived from normal human aorta [125].

Formation of glycated LDL and other advanced glycation endproducts (AGEs) enhances atherogenic potential of circulating lipoproteins that are able to induce proatherogenic lipid uptake by cultured aortic SMCs [126] and stimulate expression of RAGE and other scavenger receptor in macrophages [127]. Activation of AGE-RAGE signaling promotes vascular damage and strengthens atherosclerotic lesion progression through inducing endothelial dysfunction, attracting monocytes to the vascular intima, increasing oxidative stress, promoting vascular wall remodeling, and stimulating NF-κB-dependent expression of proinflammatory and prothrombotic molecules [128].

In diabetic patients, association of increased glycated apoB levels with elevated triglycerids, a prevalent cardiovascular risk factor [129], and myocardial infarction (MI) [130] was found suggesting for a potential prognostic value of glycated apoB for development of MI in the following five years in diabetic patients. However, Hayashi *et al.* [131] failed to show suggestive value of either glycated LDL or MDA-LDL as prognostic marker of carotid atherosclerosis in type 2 diabetic patients [131]. Further studies should be performed to precisely evaluate prognostic and diagnostic value of glycated apoB for atherosclerosis progression in diabetes. To date, size of LDL particles appears to be a better predicting marker of carotid atherosclerosis progression and stroke in type 2 diabetic subjects compared to glycated LDL [131,132,133].

### 4.4. Carbamylated LDL

Carbamylation is the reaction between isocyanic acid HNCO with amines to give urea and other carbamides [134]. Myeloperoxidase is primarily involved in carbamylation catalyzing reaction of oxidation of thiocynate to cyanate [135,136]. In the LDL particle, the apoB molecule can be carbamylated in numerous sites, preferentially in lysine residues. Chemical modification of 15% of the lysine residues in apoB-100 by carbamylation completely abolishes interaction between LDL and LDL receptor and induces switch to the scavenger receptor-mediated intake of modified LDL [137]. Levels of carbamylated LDL could be markedly increased in patients with chronic renal failure [138]. Carbamylation of proteins with urea-derived cyanate leads to renal failure and contributes to atherosclerosis, a frequent event in patients with end-stage renal disease [139]. Extensively carbamylated LDL are efficiently cleared by kidneys while the clearance rate of mildly carbamylated LDL (less than 20% of the amino groups are modified) is decreased by 2.5-fold [140]. Indeed, mildly modified LDL can accumulate in the arterial intima for prolonged time and display proatherogenic effects on vascular cells.

Carbamylated LDL are prone to further oxidation. Carbamylated-oxLDL are highly cytotoxic to endothelial cells [141,142] and induce endothelial dysfunction through stimulation of ROS-mediated signaling and activation of LOX-1 [143]. Carbamylated LDL induce proliferation of vascular SMCs [144] and increase expression of adhesion molecules intercellular adhesion molecule 1 (ICAM-1) and vascular adhesion molecule 1 (VCAM-1) thereby promoting adhesion of monocytes to endothelial cells [145]. Carbamylated LDL could be recognized by macrophage scavenger receptor A1 that mediates their intake and contributes to further cholesterol accumulation and transformation of macrophages to foam cells [135]. These LDL also promote vascular injury through enhancing oxidative stress and accelerating senescence of endothelial progenitor cells via DNA modifications and damage [146].

Carbamylated LDL are immunogenic inducing IgG autoantibodies in LDL-deficient receptor mice [147]. These antibodies are cross-reactive with oxidative-specific epitopes, especially with MDA-LDL [148] suggesting that carbamylated LDL are partially oxidized. In human plasma, proatherogenic IgG antibodies to carbamyl-LDL were also found. The antibodies were related to conditions, such as uremia and smoking, which caused increased carbalylation [147]. However, the diagnostic and prognostic value of carbamylated LDL and their self-antibodies for atherosclerosis is not evaluated yet.

### 4.5. Desialylated LDL

Sialic acid is an essential component of native LDL representing the terminal carbohydrate of biantennary sugar chains in apoB and carbohydrate chains in gangliosides [149]. Desialyation of LDL by neuraminidases, sialidase, and other glycoside hydrolases may occur naturally. Partially desialyated LDL is suggested to be subjected to further clearance from circulation by the liver [150,151]. In healthy subjects, a subfraction of electronegative LDL, which contains from 2- to 6-fold less of syalic acid compared to the native LDL, was detected [18,152,153]. This LDL subfraction was shown to be highly enriched with desialylated LDL that accounts for up to 88% of the electronegative LDL subfraction [18]. Compared to sialylated LDL, desialylated LDL particles are smaller and contain more triglycerides, fatty acids, oxysterols, and less phospholipids and antioxidants [154].

After desialylation, the monosaccharide residue (galactose) that precedes the syalic acid in the carbohydrate chain becomes terminal and externally exposed. Galactose has high affinity to lectins. Up to 80% of the electronegative LDL is bound to lectin, a phenomen allowing quantification of these LDL in blood using a lectin-sorbent assay [153] and specific isolation from blood with help of lectin-based affinity chromatography [155]. In macrophages, the lectin receptor was shown to mediate uptake of desyalylated LDL [156].

The electronegative desialylated LDL subfraction is highly proatherogenic since is able to enhance cholesterol deposits by 2- to 4-fold in cultivated normal aortic cells [157]. A minor fraction of circulating desialylated LDL (5%–10% of total LDL) could be detected in the blood of healthy subjects. In atherosclerotic patients, desialylated LDL levels are significantly increased (by 1.5- to 6-fold) compared to healthy individuals [158,159] and can account up to 60% of total LDL in CAD patients [154]. Small dense LDL particles exhibited a profound deficiency in sialylation rate correlated with increased atherogenicity of this subfraction [159]. Furthermore, desialylated LDL were shown to be more sensitive to oxidation by ROS and peroxides [160].

Tertov *et al.* [161] considered advanced desialylation of LDL as a primary step of atherogenic lipid modification. Serum sialydase may be primarily involved in enzymatic removal of sialic acid from circulating LDL [162]. In human LDL after Cu^2+^-mediated oxidation, Tanaka *et al.* [163] observed increase in content of conjugated dienes and decrease in sialylation suggesting that reactive radicals may be involved in non-enzymatic desialylation of LDL in atherosclerosis-associated oxidative stress. In desialylated LDL patricles, Tertov *et al.* [161] observed advanced loss of antioxidants along with accelerated degradation and modification of apoB with covalently bound cholesterol, a marker of lipooxidation, which increases a proatherogenic potential of modified LDL. Atherogenicity of desialylated LDL may be released through enhancing cell cholesterol intake via impairing cholesteryl ester transfer protein (CETP)-mediated reverse cholesterol transport and inhibiting esterifying activity of cholesterol acyltransferase in macrophages [164]. Under inflammatory conditions, expression of galactose-specific lectins may be up-regulated in macrophages that in turn enhances uptake of disialylated LDL by macrophages [165].

The desialylated subfraction of LDL is highly immunogenic and induces production of proatherogenic IgG self-antibodies that may contribute to atherogenesis by increasing the uptake of LDL by aortic cells [166]. Furthermore, adding exogenous anti-LDL antibodies to normal human sera results in the formation of cholesterol-containing circulating immune complexes (CICs) and induction of atherogenic properties thereby suggesting for a significant proaterogenic potential of these antibodies [89]. LDL extracted from the immune complexes represents multiple modified LDL (mmLDL) that has characteristics close or similar to those of desialylated LDL, *i.e.*, small size, higher density, higher electronegative charge, lowered content of sialic acid, increased oxysterol levels, and similar content of lipid peroxides [167,168].

## 5. Naturally Occurring Modified Forms of LDL

Certain species of oxLDL that are artificially generated *in vitro* cannot be retrieved in the blood. Despite the huge amount of work on the role of oxidized LDL in atherogenesis neither oxLDL nor MDA-LDL were detected in the blood. Indeed, LDL oxidation is likely to occur not in the blood but in the vascular wall. Circulating mmLDL were found to display signs of oxidation but oxidation is one of the various modifications occurred in the lipoprotein particle [161]. Therefore, oxLDL is a heterogeneous lipid population. Some kinds of *in vitro*-generated oxLDL cannot be retrieved in the blood. On the other hand, at least four forms of atherogenic LDL modifications were detected in the blood of patients such as small dense LDL, electronegative LDL, glycated LDL (in diabetic patients), and desialylated LDL.

Electronegative LDL [169], small dense LDL [170], desialylate LDL [157], and glycated LDL [123] isolated from the blood of patients by different groups were obtained by different methods. Naturally, the question arose what are the similarities and differences between all LDL modifications detected in the blood. It has been shown that the more electronegative LDL isolated by ion exchange chromatography is desialylated lipoprotein [153]. On the other hand, desialylated LDL has an increased electronegative charge, so it is more electronegative lipoprotein [128]. Desialylated LDL particles are smaller and possess higher density, *i.e.*, they are small dense lipoprotein particles [121]. On the other hand La Belle and Krauss [171] isolated from the blood small dense LDL that had a reduced content of sialic acid, *i.e.*, it was desialylated LDL. These data demonstrate the similarity of the two types of modified LDL. Atherogenic LDL subfraction isolated from the blood of diabetic patients represents small-dense, glycated, and desialylated lipoprotein particles [123]. These and many other features suggest that mmLDL, electronegative LDL, small dense LDL, glycated LDL, and desialylayed LDL are similar if not identical.

*Ex vivo* experiments have revealed mechanisms of multiple modification of LDL in the blood. Fraction of native LDL was isolated from blood plasma. In addition, blood serum of patients with documented atherosclerosis was obtained. LDL and serum was mixed and incubated for various periods at 37 °C. It was found that even after one-hour incubation of native LDL with atherogenic serum subfraction of desialylated LDL appears [161]. After three hours of incubation, LDL becomes able to cause accumulation of cholesterol in cultured cells. From the sixth hour of incubation with serum, LDL demonstrated reduction of neutral lipids and phospholipids as well as reduction in its size. After 36 hours of incubation, an increase in the electronegativity of the lipoprotein particles was detected. At prolonged periods of incubation (48–72 h) serum with LDL, a loss of α-tocopherol, increase of susceptibility to oxidation, and accumulation of lipid peroxidation products in LDL were observed [161]. In the same period, the degradation of apolipoprotein B in LDL begins. Thus, it has been demonstrated that modification processes making LDL atherogenic lipoprotein can occur in human blood. Desialylation of LDL particles being one of the first or primary act of modification is apparently sufficient condition for the onset of atherogenic properties. Subsequent modifications only increase the atherogenic potential of LDL. Multiple modification of LDL is a cascade of sequential changes in lipoprotein particle, namely: desialylation, loss of lipids, size reduction, increase of electronegative charge, and lipid peroxidation in LDL.

## 6. Autoantibodies against Modified LDL

### 6.1. Circulating Anti-LDL Antibodies

Modified LDL are well known to induce an adaptive immune response in atherosclerosis associated with production of self-antibodies specific to modified LDL [172]. Although circulating anti-LDL antibodies could be detected in the blood of apparently healthy non-atherosclerotic people, antibody levels are markedly increased in atherosclerosis [90]. The majority of anti-LDL antibodies detected in atherosclerotic conditions belong to the IgG class (subclasses G1 and G3) followed by IgM and IgA [173]. IgA antibodies are present in trace amounts. As mentioned above, IgG antibodies against MDA-LDL possess proatherogenic properties, while IgM antibodies are atheroprotective. The naturally circulating anti-LDL antibodies belong to the IgM class. They are responsible for specific recognizing and clearance of MDA-LDL and other modified LDL [94] and hence play the atheroprotective role. Treatment with monoclonal IgM antibodies against phosphorylcholine attenuated atherosclerosis in apoE-deficient mice [174]. In atherosclerosis, production of anti-LDL IgM antibodies could be diminished.

A spectrum of circulating anti-LDL antibodies may greatly vary depending on the pathological conditions. For example, the majority of IgG fraction of anti-LDL antibodies isolated from blood of type 2 diabetic patients were specific to MDA-LDL (>70%) followed by anti-AGE-LDL antibodies. Trace levels of antibodies against carbamyl-LDL, LDL modified by myeloperoxidase-dependent oxidation, and (hexanoyl) lysine-LDL (a product of reaction of linoleic acid hydroperoxide and lysine) could be also detected [175].

Anti-LDL antibodies specific to various types of modified LDL may be cross-reactive between each other suggesting for presence of shared epitopes. For example, AGE-LDL antibodies that are primarily reactive with AGE-LDL show cross-reactivity with MDA-LDL and carbamyl-LDL due to the presence of carbamylated lysine epitopes in MDA-LDL [176].

Detection of antibodies against MDA-LDL is often regarded as evidence of the existence of oxLDL *in vivo*. In blood, anti-LDL autoantibodies were first detected by Palinski *et al.* [177]. The authors [177] have established that the anti-LDL antibodies were specific for MDA-LDL. They have presented the discovery of self-antibodies against MDA-LDL as a proof that that oxidized LDL exist *in vivo* [177]. Somewhat later, in the blood of patients with atherosclerosis self-antibodies against modified LDL have been found, identified and described [166,178]. Moreover, affinity of these antibodies to different forms of lipoprotein modification was evaluated [178] (Table 1). Lipoprotein modified by glycosylation, acetylation and copper-oxidation reacted with autoantibody with the same affinity as native LDL from healthy subjects. LDL isolated from the blood of patients with assessed atherosclerosis (mixture of multiply-modified and native LDL) reacted with anti-LDL affinity with an order higher (Table 1).

It was found that also MDA-LDL reacts with anti-LDL with similarly high affinity as LDL from patients. Thus, it was confirmed that affinity of autoantibodies to MDA-LDL is higher compared to that to native LDL. However, autoantibodies had the highest affinity to desialylated LDL. Affinity constant of autoantibodies to desialylated LDL was an order higher than to MDA-LDL and two orders higher than to native LDL (Table 1). Thus, anti-LDL antibodies that are primarily react with desialylated LDL show cross-reactivity with MDA-LDL. Obviously, autoantibodies are produced in response to the appearance of desialylated LDL but not oxLDL.

**Table 1:** Affinity constants of anti-low-density lipoproteins (LDL) (×10^−7^·М^−1^). Adapted with permission from [166]. (1991) (Orekhov, A.N.; Tertov, V.V.; Kabakov, A.E.; Adamova, I.Yu.; Pokrovsky, S.N.; Smirnov, V.N.).

LDL Preparation	Affinity Constant (×10^−7^·М^−1^)
LDL from healthy subjects	2.4
glycosylated LDL	2.6
acetylated LDL	2.8
Cu^2+^-oxidized LDL	3.5
LDL from atherosclerotic patients	11.3
MDA-LDL	10.9
Desialylated LDL	89.4

### 6.2. Diagnostic and Prognostic Value of Anti-LDL Self-Antibodies

Circulating self-antibodies against LDL can be useful as diagnostic and prognostic markers of cardiovascular risk. Doo *et al.* [179] showed that MDA-LDL antibodies can have a predictive value for cardiac events in patients with unstable angina pectoris. Increased titers of MDA-LDL IgG showed association with elevated expression of C-reactive protein and adhesion molecules and may suggest for plaque instability in angina pectoris [179]. Similarly, a role of increased levels of MDA-LDL antibodies as a predictor of atherosclerotic complications such as acute coronary syndrome in patients with vulnerable lesions was shown in other studies [180,181].

For MDA-LDL antibodies, in a large prospective epidemiologic European Prospective Investigation into Cancer (EPIC)-Norfolk Study including non-selected and initially healthy population, levels of IgG and IgM antibodies were shown to predict risk of CAD events but this risk is modulated by oxidative markers. Anti-MDA-LDL antibodies showed an inverse correlation with CAD events suggesting for their atheroprotective role [182]. The atheroprotective role of MDA-LDL antibodies in carotid atherosclerosis was shown by Karvonen *et al.* [183].

However, there are studies that do not support the prognostic value of anti-LDL antibodies for cardiovascular events. The significant discrepancy in results can be explained by difference in selection criteria used to recruit patients, heterogeneity of groups of patients tested, difference in protocols used to purify antibodies, lack of standardization in antibody-detecting immunoassays, small numbers of patients studied, *etc.* [90]. In fact, each subfraction of antibodies specific to a certain type of modified LDL represents pool of polyclonal antibodies reacting with different modified epitopes in the LDL particle.

The diagnostic value of anti-oxLDL antibodies in atherosclerosis is seriously limited by several obstacles. The lack of standard protocols in measuring serum titers of anti-oxLDL antibodies is likely to represent the major reason of inconsistent results produced by different groups [184]. Ox-LDL generated *in vitro* by copper oxidation or with help of horseradish peroxidase (HRP) are commonly used as antigens to induce antibody production. However, anti-copper-oxLDL antibodies displayed no cross-reactivity with anti-HRP-oxLDL-antibodies [185] that could generate discrepancy in quantification of those antibodies. Some circulating ox-LDL antibodies are naturally present. For example, in apoE-deficient mice, natural protective IgM antibodes against oxidized phospholipids of *Streptococcus pneumoniae* were detected [186] and measurement of these atherosclerosis-unrelated antibodies could aberrantly increase the total titer of oxLDL antibodies. Furthermore, natural IgM antibodies recognizing oxidized epitopes are widely present in mice and humans and play a marked role in host immunity and clearance of apoptotic cells [187]. Finally, extensive formation of immune complexes between anti-LDL antibodies and oxLDL could significantly decrease serum levels of free anti-oxLDL antibodies, especially in patients with autoimmune disease such as type 1 diabetes [188,189].

Anti-LDL antibodies can indeed form immune complexes with modified LDL that circulate in the blood. Interestingly, MDA-LDL antibodies isolated from the immune complexes (*K*_d_ = ~10^−8^ mol/L) had higher affinity than free MDA-LDL antibodies circulating in the bloodstream (*K*_d_ = ~10^−7^ mol/L) [175]. Indeed, antibodies that are able to form circulating immune complexes (CICs) are more specific to modified LDL. In addition, anti-LDL antibodies that present in the immune complexes may interfere with the assay of anti-LDL antibodies [189,190]. Indeed, levels of LDL-containing CICs may better correlate with atherosclerosis progression than circulating anti-LDL antibodies [91].

## 7. LDL-Containing Circulating Immune Complexes

### 7.1. Atherogenic and Proinflammatory Properties of LDL-Containing Immune Complexes

**Figure 1:**
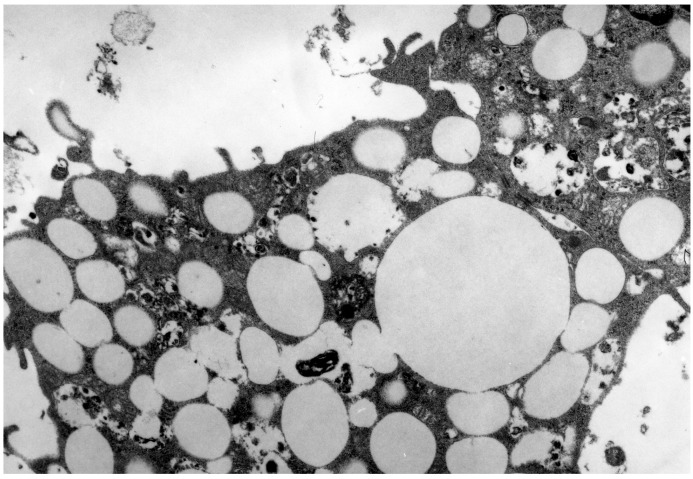
Lipid-filled vacuoles occupy the most portion of the cytoplasm of macrophages incubated with lipoprotein-containing CICs. Electron microscopy. Magnification: ×7800.

The atherogenicity of immune complexes containing modified LDL was first demonstrated by Klimov *et al.* [191], who observed a 60-fold increase in accumulation of cholesterol esters in murine peritoneal macrophages incubated with lipoprotein-containing CICs prepared *in vitro* from radiolabeled LDL and anti-apoB IgG. Morphologically, macrophages incubated with lipoprotein-containing CICs display an appearance of typical foam cells (Figure 1). Treatment of macrophages with lipoprotein immune complexes isolated from human atherosclerotic sera resulted in an almost three-fold increase in cholesterol ester deposits [192]. These data were then independently confirmed [89,193,194].

Fc receptors including FcγRI and FcRIIa were shown to mediate intake of LDL-containing CICs by human macrophages [195,196,197]. The Fc receptor-mediated intake of the immune complexes leads to the proinflammatory activation of monocytes/macrophages, formation of foam cells, and release of proinflammatory cytokines such as tumor necrosis factor (TNF)-α and interleukin (IL)-1β [198]. In endothelial cells, immune complexes induce expression of inflammatory chemokines IL-8 and monocyte chemoattractant protein-1 (MCP-1) [199]. OxLDL-IgG CICs are able to initiate proinflammatory response in cultured human mast cells by up-regulating expression of TNF-α, IL-8, and MCP-1 [200].

It should be noted that proatherogenic and proinflammatory effect of LDL-containing CICs on human macrophages/monocytes is stronger than that of modified LDL. Incubation of cultured macrophages with the immune complexes resulted in more profound accumulation of cholesterol esters [89,201] and more potent production of TNF-α, IL-1β, IL-6, and other proinflammatory cytokines [202]. In macrophages, oxLDL rapidly stimulated activity of lysosomal sphingomielinase that then declines below baseline. In contrast, LDL-containing CICs caused prolonged and consistent activation of lysosomal and secretory sphingomielinase that regulates release of proinflammatory exosomes containing heat shock protein Hsp70 and IL-10 [203]. Hammad *et al.* [204] found that treatment of resulted in induction of a network of genes involved in stress response, endocytosis, regulation of expression, protein and lipid transport, and inflammation including activation of NF-κB and cytokine production by monocytes. Finally, MDA-LDL-containing CICs were shown to induce increased release of matrix proteinases by macrophages suggesting on possible involvement in plaque vulnerability and acute coronary syndrome [205].

### 7.2. Diagnostic and Prognostic Value of LDL-Containing Immune Complexes

In 1990, Orekhov *et al.* [206] and Tertov *et al.* [207] first described determining of the content of cholesterol presented in precipitated immune complexes as a surrogate for LDL. The authors showed that content of both cholesterol and apoB in the complexes is well correlated with the atherogenicity of human serum containing these complexes [206,207]. It was suggested that atherogenic potential of CAD sera can be attributed to the presence of the immune complexes [207]. The atherogenicity of LDL-containing CICs was then replicated by other groups [208,209] that reported on the accumulation of cholesterol esters in macrophages mediated by LDL-containing CICs isolated from sera of patients. The cholesterol accumulation was significantly correlated with the content of cholesterol, apoB, IgG, and IgA in isolated complexes.

The prognostic value of total cholesterol presented in the immune complexes as a surrogate biomarker of atherosclerosis was confirmed by Lopes-Virella *et al.* [189] in a small follow-up study involved 49 patients with type 1 diabetes. In an eight-year follow-up period, the patients developed CAD. Lopes-Virella *et al.* [189] showed negative correlation between the total cholesterol in polyethylene glycol (PEG) precipitates used as a surrogate measurement of oxLDL-containing CICs precipitated by PEG and concentration of free oxLDL antibodies. The oxLDL-containing CICs were considered as a risk factor for macrovascular complications in type 1 diabetes [189].

**Figure 2:**
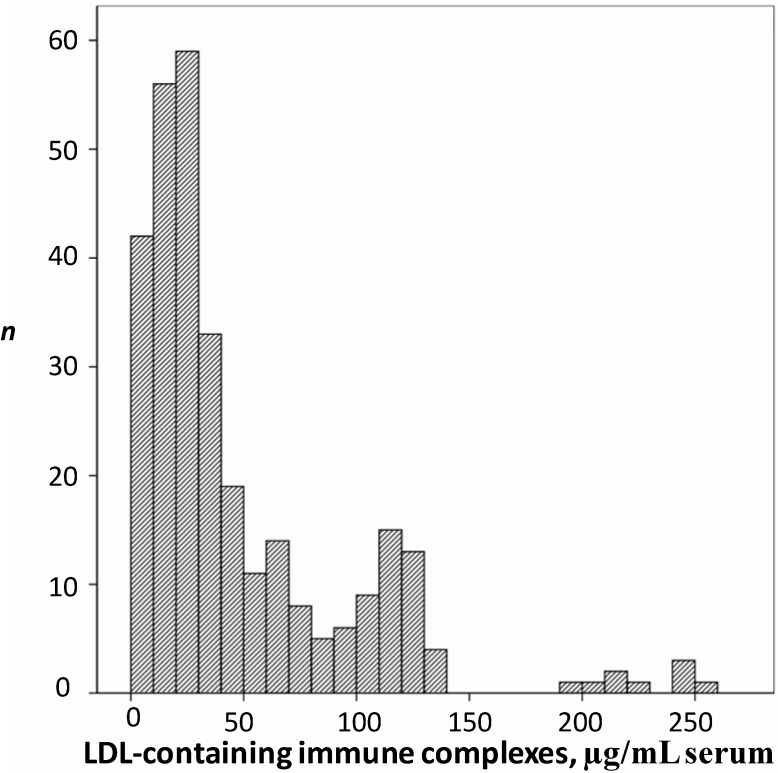
The frequency distribution of LDL-containing circulating immune complexes (CICs) depending on their levels in sera of 318 apparently healthy men (aged 40–78) asymptomatic for ischemic heart disease. The serum content of LDL-containing CICs (μg/mL serum) is shown in the horizontal axis. The vertical axis shows the number of observations (*n*).

The role of cholesterol content in the immune complexes as a predictive marker for carotid artery atherosclerosis and five-year carotid IMT progression in type 1 diabetes was then showed in the large prospective Epidemiology of Diabetes Interventions and Complications (EDIC)/Diabetes Control and Complications Trial (DCCT) comprising 1050 diabetic individuals [210]. Recently, in a Two-year follow-up study, Sobenin *et al.* [211] reported relation of elevated levels of cholesterol and LDL associated with the immune complexes with increased carotid IMT suggesting the prognostic value of these lipid parameters for progression of carotid atherosclerosis. The presence of the normal level of LDL-containing CICs (<16.0 μg/mL) was shown to predict the lack of carotid atherosclerosis progression for the next two years at prognostic value of 78.3% (Figure 2) [212].

Quantification of LDL subfractions in the circulating immune complexes derived from sera of 473 type 1 diabetic patients revealed strong association of MDA-LDL and AGE-LDL with IMT after adjustment for conventional risk factors suggesting for a robust predictive value of both parameters for progression of carotid atherosclerosis in type 1 diabetes [213,214]. Furthermore, the content of MDA-LDL in the immune complexes was found to be independently associated with the coronary artery calcification score indicating significance of oxLDL levels in the immune complexes as an independent risk marker of arterial calcification (RR = 1.23) in type 1 diabetes [215]. However, in type 2 diabetes (the Veterans Administration Diabetes Treatment (VADT) Study), MDA-LDL presented in the immune complexes showed a better predictive value for MI (Hazard Risk = 2.44 for patients at the highest quartile of the MDA-LDL content in the immune complexes to develop MI *vs.* the patients at the lowest quartile) compared with MDA-LDL and AGE-LDL [216]. The significance of the MDA-LDL content as a predictive marker for progression to MI can be explained by the involvement of MDA-LDL in the control of plaque stability/rupture [217]. MDA-LDL could contribute to the development of vascular injury in atherosclerosis by inducing arterial denudation through cytotoxic effects on vascular endothelium [218].

### 7.3. Diagnostic and Prognostic Potential of Multiple Modified LDL

**Figure 3:**
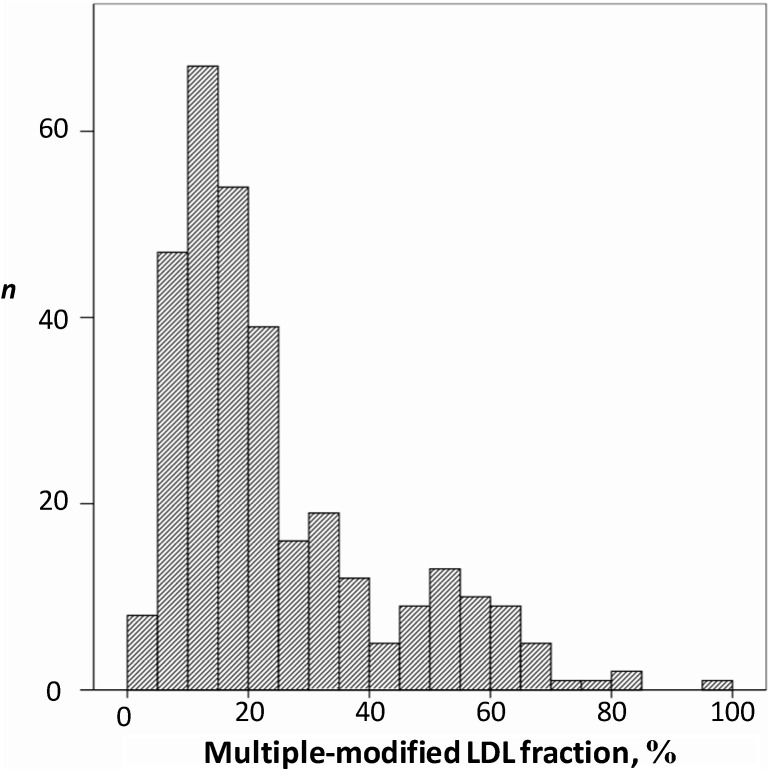
The frequency of distribution of multiple-modified LDL (mmLDL) depending on their subfraction content in total LDL from sera of 318 men (aged 40–78) asymptomatic for ischemic heart disease. The percentage of mmLDL fraction in total serum LDL is presented in the horizontal axis. The vertical axis displays the number of observations (*n*).

An open-label cross-sectional study was performed in 330 patients, men and women aged 45–78, to establish the relationship between novel lipid parameters (mmLDL, LDL-containing circulating immune complexes, and the ability of serum to induce intracellular lipid accumulation) in atherosclerosis. On the basis of clinical and laboratory examination, the study participants were divided into three groups: asymptomatic low-risk study participants (*n* = 58), hypercholesterolemic coronary heart disease (CHD)-free study participants with serum cholesterol above 250 mg/dL (*n* = 134), and the patients with clinically manifested atherosclerosis in the form of CHD and/or personal history of myocardial infarction (*n* = 138). The interquartile range of proportion of mmLDL accounted for 10%–25% of a total serum apoB-containing LDL (Figure 3).

The direct correlation was found between the proportion of mmLDL and the ability of serum to induce lipid accumulation in cultured blood-derived monocyte-macrophages (*r* = 0.274, *p* < 0.001). It is notable that the correlation was more pronounced than in the case of LDL-containing CICs [155,211,212]. The correlation between the proportion of mmLDL and the level of LDL-containing circulating immune complexes (*r* = 0.349, *p* < 0.001) was also revealed in this study. These results were considered as the indication of the role of mmLDL both in formation of serum atherogenic potential and LDL-CICs. The two-fold increase in proportion of multiple modified LDL was observed in hypercholesterolemic patients as compared to low-risk study participants (*p* < 0.001); in patients with clinically manifested atherosclerosis the proportion of mmLDL was comparable to low-risk study participants, possibly due to the effects of intensive therapeutic interventions.

The results of this study suggest that the diagnostic and prognostic significance of mmLDL is at least not lower than that of LDL-containing CICs.

## 8. Conclusions

In atherosclerosis, blood LDL are subjected to multiple enzymatic and non-enzymatic modifications that increase their atherogenicity and induce immunogenicity [154,166,172]. Modified LDL are capable to induce vascular inflammation through activation of innate immunity that contributes to progression of atherogenesis. The immunogenicity of modified LDL results in induction of self-antibodies specific to a certain type of modified LDL. The antibodies react with modified LDL forming circulating immune complexes. In fact, up to 90% of modified LDL in circulation exist as constituents of the immune complexes [214].

Circulating immune complexes exhibit prominent immunomodulatory properties that influence atherosclerotic inflammation and atherogenesis itself. Compared to freely circulating modified LDL, modified LDL associated with the immune complexes have a more robust atherogenic and proinflammatory potential. Importantly, various lipid components of the immune complexes may serve, not only as diagnostic, but also as essential predictive markers of cardiovascular events in atherosclerosis [172,211,212]. It should be stressed that Lopes-Virella and collaborators observed significant association between both total LDL particle levels [219] and the LDL content in the circulating immune complexes [189] with carotid IMT in type 1 diabetes. Regardless of LDL size, increased LDL levels were positively associated with the LDL content in the immune complexes [220]. Indeed, formation of LDL-containing immune complexes may provide a physiological link and explain whereby elevated LDL levels contribute to macrovascular disease in type 1 diabetes. Similar experiments should be performed in atherosclerotic patients and type 2 diabetic patients in order to explain predictive significance of LDL-containing immune complexes in cardiovascular pathology.

In conclusion, the accumulating evidence indicates that the quantification of modified LDL associated with immune complexes has a predictive value superior of that of traditional risk markers that are currently in use [172,211,212].
